# Peritoneal resident macrophages in tumor metastasis and immunotherapy

**DOI:** 10.3389/fcell.2022.948952

**Published:** 2022-08-11

**Authors:** Yu Zhang, Dongyun Ouyang, Youhai H. Chen, Houjun Xia

**Affiliations:** ^1^ Center for Cancer Immunology, Institute of Biomedicine and Biotechnology, Shenzhen Institute of Advanced Technology, Chinese Academy of Sciences, Shenzhen, China; ^2^ Department of Immunobiology, College of Life Science and Technology, Jinan University, Guangzhou, China

**Keywords:** resident macrophage, peritoneal metastasis, tumor immunity, immunotherapy, ontogeny, immune evasion, tumor microenvironment, tumor associated macrophage

## Abstract

Macrophages residing in various tissues play crucial roles in innate immunity, tissue repair, and immune homeostasis. The development and differentiation of macrophages in non-lymphoid tissues are highly regulated by the tissue microenvironment. Peritoneum provides a unique metastatic niche for certain types of tumor cells. As the dominant immune cell type in peritoneal cavity, macrophages control the immune response to tumor and influence the efficacy of anti-tumor therapy. Considering the heterogeneity of macrophages in origin, metabolism, and function, it is always challenging to define the precise roles of macrophages in tumor microenvironment. We review here recent progresses in peritoneal resident macrophage research in the context of physiological and metastatic tumor conditions, which may benefit the development of new anti-tumor therapies through targeting macrophages.

## 1 Introduction

Macrophages are critical for immunity, tissue repair, and organ regeneration. Tissue resident macrophages are observed in multiple tissues and defined as a population genetically and developmentally distinct from inflammatory macrophages (Cox et al., 2021). Unlike blood monocytes or differentiated inflammatory macrophages, tissue resident macrophages maintain their population in-site *via* self-renewal without the contribution of blood monocytes and hardly move to other tissues (Yona et al., 2013; Dick et al., 2019). Genetic fate-mapping strategies in mouse developmental model indicates most of the tissue resident macrophages originate from colony-stimulating factor 1 receptor (*Csf1r*)-expressing erythromyeloid progenitors (EMPs) which arise at approximately embryonic day 8 (E8) from the yolk sac hemogenic endothelium (Gomez Perdiguero et al., 2015). At about E10, hematopoietic stem cells (HSCs) migrate into the fetal liver, which serves as the major organ of HSC differentiation until birth (Gekas et al., 2005). Meanwhile, HSCs progenitors replenish part of tissue resident macrophages under physiological and pathological contexts (Hashimoto et al., 2013; Bain et al., 2014). Although tissue resident macrophages in different tissues share certain core activities, they display functional diversity to support the homeostasis of each tissue. Dysfunction of tissue resident macrophages causes severe and often fatal developmental disorders as reported in recent experimental works in mice (De Schepper et al., 2019; Oosterhof et al., 2019). Understanding their diversity and contribution to pathophysiological processes may provide new therapeutic targets for human diseases, especially tumor.

Tumorigenesis is mediated by mutated genes which not only support uncontrolled tumor cell growth but also protect them from immune surveillance. Tumor associated macrophages (TAMs) represent the dominant myeloid cell population in most types of solid tumors of both humans and mice. TAMs contribute to the immunosuppressive tumor microenvironment (TME) which protects tumor cells from anti-tumor immune response (Pittet et al., 2022). However, the connection between tissue resident macrophages and TAMs in different solid tumors is still not well understood. Monocytes have been considered as the precursors of TAMs for many years since CCL2/CCR2 signaling which recruits monocytes from bone marrow to tumor facilitates breast cancer metastasis (Qian et al., 2011). Furthermore, detailed research points out monocyte-derived macrophages, but not mammary tissue macrophages, promote the breast cancer development (Franklin et al., 2014). Recently, new research revealed the pro-tumoral role of tissue resident macrophages, similar to monocytic macrophages, in tumorigenesis and tumor growth among certain types of tumors. For example, the pancreatic tissue resident macrophages originate from embryonic hematopoiesis and promote pancreatic ductal adenocarcinoma (PDAC) progression (Zhu et al., 2017). Consistently, the lung resident interstitial macrophages contribute to the pool of TAMs together with CCR2-dependent recruited macrophages (Loyher et al., 2018). Moreover, we and other groups demonstrate that the peritoneal resident macrophages promote ovarian cancer development and metastasis into peritoneal cavity (Casanova-Acebes et al., 2020; Etzerodt et al., 2020; Xia et al., 2020). Therefore, the involvement of tissue resident macrophages may decide the fate of tumor progression.

Peritoneal cavity is a fluid-filled space located between the wall of the abdomen and the organs found in the abdomen. Peritoneal resident macrophages are well-studied as mouse primary macrophages in peritoneal cavity and share very similar characters with macrophages obtained from both pleural cavity and pericardial cavity. Peritoneal resident macrophages maintain serosal homeostasis and provide immune surveillance in the etiology of pathologies including peritonitis, endometriosis, and metastatic cancers. Here, we summarize the new concepts related to the development and differentiation of cavity-resident macrophages, especially peritoneal resident macrophages, and their roles in tumor metastasis and immunotherapy.

## 2 The basic biology of peritoneal resident macrophages in steady-state mouse

### 2.1 The phenotypes of peritoneal resident macrophages

Peritoneal macrophages have been used as the source of primary mouse resting macrophages for almost 60 years since Cohn and collaborators started to collect and analyze them in the 1960s (Steinman and Moberg, 1994). At that time, these cells were considered as an individual macrophage population since they are characterized by the classical mouse macrophage markers CD11b and F4/80. Further, peritoneal resident macrophages have been divided into large peritoneal macrophages (LPMs) and small peritoneal macrophages (SPMs) based on their size (Ghosn et al., 2010). In the meantime, the two subsets display phenotypic differences that LPMs have been found to be F4/80^High^MHC-II^Low^ while SPMs to be F4/80^Low^MHC-II^High^. The LPMs are the long-lived macrophages since they express more mature markers as CD40, CD80, and CD86 (Ghosn et al., 2010). Some unique markers are specifically expressed on each subsets, for example T-cell immunoglobulin and mucin domain containing 4 (TIM4) and Intercellular adhesion molecule 2 (ICAM2) expressed on LPMs while CCR2 and CD226 expressed on SPMs (Kim et al., 2016) (Table 1). In steady-state mouse, LPMs are the dominant population (more than 90%) with very identical profiles while SPMs are a very small (less than 10%) and heterogeneous population consisted with several monocytic subsets (Bain et al., 2016; Kim et al., 2016).

**TABLE 1:** The phenotypic difference between LPMs and SPMs in steady-state mice.

Surface markers	Peritoneal resident macrophages (LPMs)	Monocyte derive macrophages (SPMs)	References
CCR2	−	+	(Kim et al., 2016; Xia et al., 2020)
CD9	High	Low	Rosas et al. (2014)
CD11b	High	Low	(Ghosn et al., 2010; Gautier et al., 2012)
CD11c	+	+/−	(Ghosn et al., 2010; Bain et al., 2016)
CD24	+	−	Rosas et al. (2014)
CD40	High	Low	Ghosn et al. (2010)
CD49f	+	−	Okabe and Medzhitov, (2014)
CD64	+	−	Gautier et al. (2012)
CD73	+	−	(Okabe and Medzhitov, 2014; Rosas et al., 2014)
CD80	High	Low	Ghosn et al. (2010)
CD86	High	Low	Ghosn et al. (2010)
CD93	+	−	(Okabe and Medzhitov, 2014; Rosas et al., 2014)
CD102 (ICAM2)	+	−	(Okabe and Medzhitov, 2014; Kim et al., 2016; Bain et al., 2020)
CD206	−	+	Accarias et al. (2016)
CD209b	High	Low	Rosas et al. (2014)
CD226	−	+	Kim et al. (2016)
CRIg	+	−	(Rosas et al., 2014; Xia et al., 2020)
F4/80	High	Low	(Ghosn et al., 2010; Gautier et al., 2012; Okabe and Medzhitov, 2014; Bain et al., 2016)
MerTK	+	−	Gautier et al. (2012)
MHC-II	Low	High	(Ghosn et al., 2010; Okabe and Medzhitov, 2014; Bain et al., 2016; Kim et al., 2016)
TIM4	+	−	(Rosas et al., 2014; Bain et al., 2016; Xia et al., 2020)
TLR4	High	Low	Ghosn et al. (2010)

Level of expression: + positive; − negative.

### 2.2 The determinants of peritoneal resident macrophage differentiation

**FIGURE 1:**
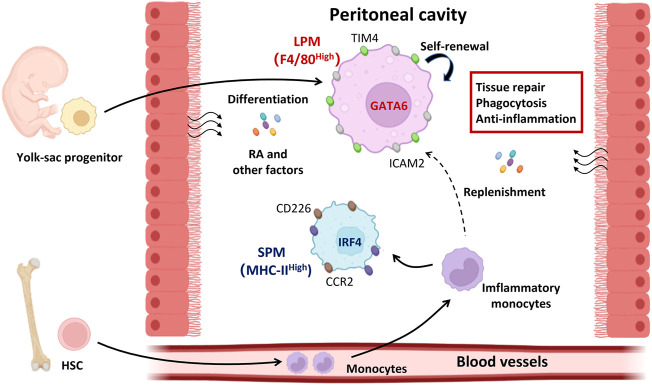
The determinants of peritoneal resident macrophage differentiation. Under steady state, both LPMs and SPMs represent the peritoneal resident macrophages. LPMs are dominant in the peritoneal cavity and function in tissue repair, phagocytosis, and anti-inflammation. LPMs are differentiated from the yolk-sac progenitors and maintain their numbers by self-renewal. Local environment supports the LPMs differentiation by secreting the retinoic acid and other factors. Different from LPMs, SPMs are differentiated from HSC derived monocytes and expand themselves in peritoneal cavity once inflammation occurs. The differentiation of LPMs is mediated by transcriptional factor GATA6 while that of SPMs is IRF4. GATA6, GATA binding protein six; HSC, hematopoietic stem cells; IRF4, interferon regulatory factor 4; LPMs, large peritoneal macrophages; RA, retinoic acid; SPMs, small peritoneal macrophages.

Several critical factors determine the formation of macrophage niches which control the size of the macrophage population and imprint their tissue-specific identity in peritoneal cavity, including the ontogeny, intrinsic factors, and local environment (Figure 1).

#### 2.2.1 Ontogeny

It is important to understand how the tissue macrophages establish their compartment during mammalian development and aging. The population of tissue resident macrophages in different location and at different time points can arise from three distinct waves of precursors: early yolk sac macrophages, fetal liver monocytes, or bone marrow derived monocytes (Bleriot et al., 2020). In the steady state condition, most tissue resident macrophages in mice and humans originate from the embryonic stage and are maintained by self-renew but not differentiated from adult hematopoiesis (Ginhoux and Guilliams, 2016). Indeed, the fate-mapping studies demonstrate the precursors of LPMs migrate and reside in the peritoneal cavity during embryonic stage and are shaped by the tissue microenvironment (Yona et al., 2013; Bain et al., 2016). In the young mice, embryonic derived LPMs depend on self-proliferation to sustain their numbers. However, bone marrow derived macrophages can differentiate and acquire key characteristics of the embryonic population in peritoneal cavity and finally replenish the embryonic derived LPMs with aging (Bain et al., 2016).

#### 2.2.2 Intrinsic factors

In addition to ontogeny, intrinsic factors also have an important effect on the peritoneal macrophage differentiation, such as genetic background, sex difference, and phagocytosis. Systematic genetic and epigenetic analyses of tissue resident macrophages from five diverse strains of mice discovered the macrophage identity was linked to more than 100 transcription factors that in turn bind to hundreds of connected cis-regulatory domains (Link et al., 2018). Therefore, mutation of these regulatory elements might disturb the specific macrophage identity. For example, deletion of a specific enhancer of *Csf1r* gene, the fms-intronic regulatory element (FIRE), selectively affects peritoneal resident macrophage populations (Rojo et al., 2019). In addition, CCAAT/enhancer binding protein (C/EBP)-β, functioning as macrophage restricted lineage determining transcription factor, plays an intrinsic role in the generation of LPMs (Cain et al., 2013). Sex differences modulate the immune response (Wilkinson et al., 2022). Sex has been proposed to affect brain microglia differentiation (Thion et al., 2018). Similarly, peritoneal resident macrophages exhibit sexually dimorphic traits that their replenishment from the bone marrow is higher in males compared to that in females, which is driven by changes in the local microenvironment that arise upon sexual maturation (Bain et al., 2020). Phagocytosis, the phagocytes restricted activity to uptake the particles discovered by Ilya Metchnikoff in starfish larvae in 1880s (Epelman et al., 2014), transiently decides the resident macrophage identity by polarizing the cell toward an anti-inflammatory phenotype (A-Gonzalez et al., 2017). But the abilities of phagocytosis between LPMs and SPMs are not the same. At the early stage of infection, LPMs are the major bacterial phagocytic cells beyond SPMs (Ghosn et al., 2010). Consistently, it is convinced in the context of apoptotic cells clearance by peritoneal and pleural resident macrophages which is shown the transcription factors KLF2 and KLF4 initiate the apoptotic cell clearance program in tissue residential macrophages (Roberts et al., 2017). This type of program in peritoneal resident macrophages is context dependent and reversible.

#### 2.2.3 Local microenvironment

The local microenvironment, so-called “niche of residence” (Guilliams and Scott, 2017), provides signals necessary for the maturation of functional resident macrophages from any precursor in a time-dependent manner. The transcriptional factor GATA binding protein 6 (GATA6) is responsible for the development and identity of peritoneal resident macrophage, specifically expressed in LPMs but not SPMs (Gautier et al., 2012; Gautier et al., 2014; Okabe and Medzhitov, 2014; Rosas et al., 2014). Retinoic acid, a metabolite of vitamin A, induces tissue-specific localization and functional polarization of LPMs through the induction of GATA6 expression (Gautier et al., 2014; Okabe and Medzhitov, 2014). The omentum which is formed by a double layer of mesothelial cells in peritoneal cavity provides continuous retinoic acid and other factors to support the LPMs profiles (Okabe and Medzhitov, 2014). Furthermore, transcription factor Wilms’ tumor 1 (WT1) positive mesothelial and fibroblastic stromal cells induce retinoic acid-dependent and -independent hallmark genes of GATA6^+^ macrophages by expressing two rate-limiting enzymes, aldehyde dehydrogenases (ALDH)-1 and -2, in retinol metabolism (Buechler et al., 2019). Hence, serous cavity restricts the peritoneal resident macrophage identity.

### 2.3 The homoeostasis of peritoneal resident macrophages

In newborn mouse, LPMs expand *via* local proliferation which is considerably reduced and most likely provides homeostatic control of cell numbers in the adult (Davies et al., 2011). The proliferative capacity of LPMs is determined by signals from the local microenvironment rather than their genetic heterogeneity and origin (Bain et al., 2016; Bain et al., 2020). CSF1-Fc or IL-4c is known to drive proliferation of peritoneal macrophages in steady state or TH-2 inflammation (Jenkins et al., 2011; Jenkins et al., 2013). During the resolution of inflammation, LPMs survive and then undergo a transient and intense proliferative burst *in situ* to repopulate the tissue which is M-CSF dependent and highly decided by the expression of GATA6. Selective GATA6 deficiency resulted in dysregulated peritoneal macrophage proliferative renewal during homeostasis and in response to inflammation, which was associated with delays in the resolution of inflammation (Rosas et al., 2014). In addition, GATA6-deficient macrophages are vulnerable to death and lead to a reduction of peritoneal resident macrophages by lacking functional aspartoacylase (Gautier et al., 2014). Accordingly, mammalian target of rapamycin complex 2 (mTORC2) has been found negatively regulate the GATA6 expression by controlling forkhead box O1 (FOXO1) activation. Hence, mTORC2 deficiency enhances the generation of tissue-resident peritoneal cells through increased proliferation and cell survival (Oh et al., 2017).

Notably, the proliferation activity of peritoneal macrophage displays a sexual dimorphism that LPMs proliferate faster at the resolution stage of inflammation in male mice than that in female mice. Consistently, the signature of male peritoneal macrophages was dominated by cell cycle-associated genes compared to genes associated with lipid uptake and transport as well as immune response in female (Bain et al., 2020). Interestingly, peritoneal macrophages express estrogen receptors which mediated enhanced proliferation in response to exogenous estrogen. Furthermore, ovariectomy leads to a reduction in the number of macrophages which imply the sex maturation influences the biology of the peritoneal resident macrophages (Pepe et al., 2017).

### 2.4 The metabolic profiles of peritoneal resident macrophages

The metabolism of macrophage is highly linked with their activation, polarization, and function (Leone and Powell, 2020). Macrophages in tissue establish metabolic adaptation to support homeostatic tissue function and facilitate wound healing. The peritoneal resident macrophages express the GATA6 associated gene *Aspa*, which encodes the hydrolase enzyme aspartoacylase to catalyze the deacylation of N-acetylaspartate into aspartate and acetate (Gautier et al., 2014). The peritoneal cavity is enriched for N-acetylaspartate relative to its abundance in serum. Considering the acetate is the precursor of acetyl-CoA which has been used as fuel for the tricarboxylic acid (TCA) cycle, peritoneal resident macrophages displays higher level of mitochondrial oxygen-consumption rates compared with that in bone marrow derived macrophages *in vitro* (Davies et al., 2017). In addition, the concentration of glutamate is higher in the peritoneal cavity than in serum which is able to supplement glutamine to maintain respiratory burst during phagocytosis *via* enhancing mitochondrial complex-II metabolism in peritoneal resident macrophages (Davies et al., 2017). Consistently, peritoneal resident macrophages increase TCA cycle-associated genes and mitochondrial mass compared with monocyte derive macrophages. Inhibition of mTORC2 increases TCA cycle-associated genes expression and mitochondrial mass which may be due to the elevated GATA6 expression (Oh et al., 2017). Hence, the diversity of metabolites in peritoneal cavity may determine the metabolic profiles through modulating GATA6 expression in peritoneal resident macrophage.

## 3 The role of peritoneal resident macrophages in tumor metastasis into the cavity

**FIGURE 2:**
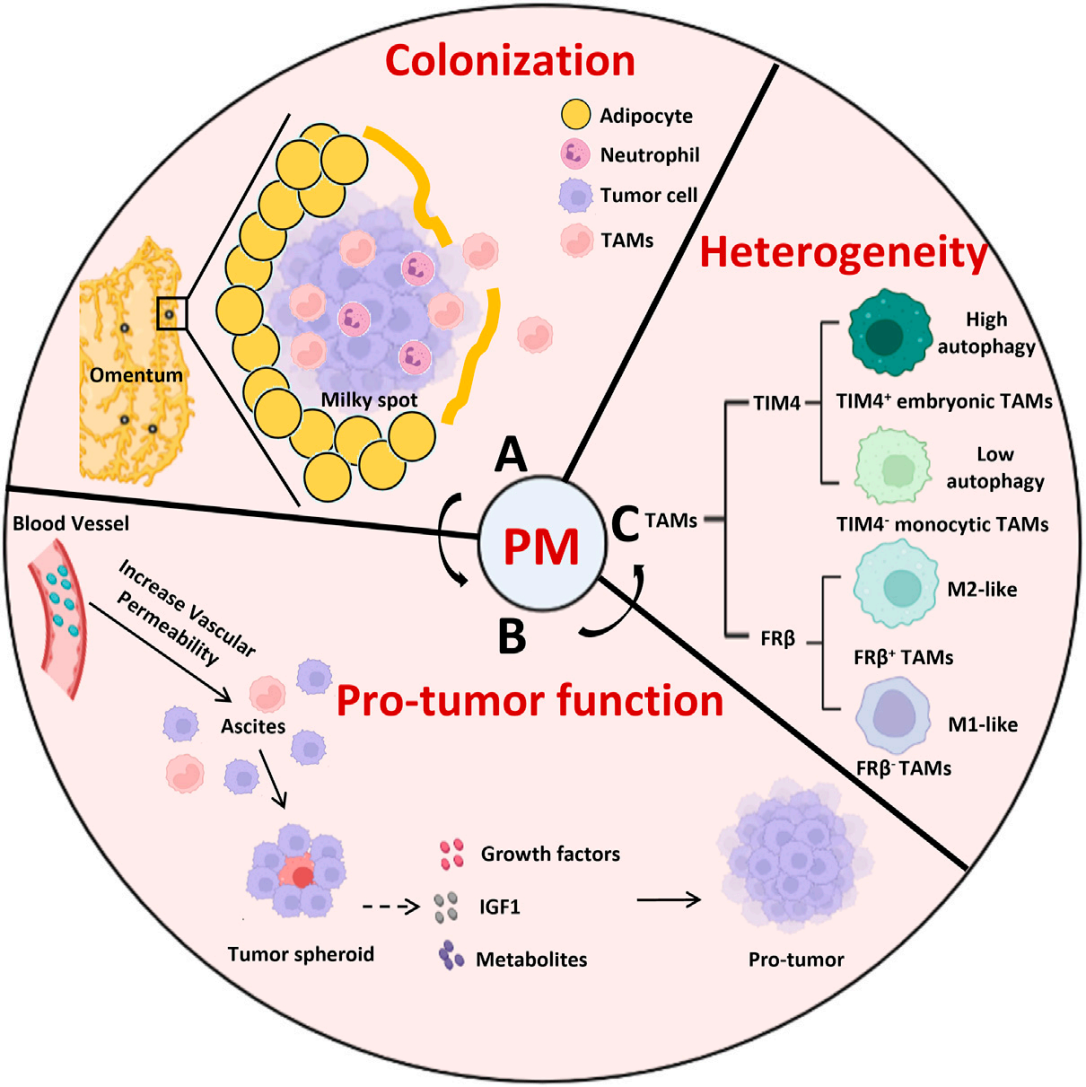
The role of peritoneal resident macrophages in the tumor metastasis into the cavity. **(A)** The colonization of omentum is a critical step for tumor metastasis, which is occurred passively through the milky spots. **(B)** TAMs promote the tumor peritoneal metastasis through gathering tumor cells to form the spheroid and secreting growth factors, IGF1, metabolites to support the tumor growth. **(C)** The heterogenicity of TAMs distinguished by TIM4 or FRβ in peritoneal metastasis. Compared to TIM4^-^ monocytic TAMs, TIM4^+^ embryonic TAMs express more OXPHOS genes and adapt to autophagy to support their survival. FRβ^−^ TAMs display a round shape that was more monocytic in appearance and consistent with M1 phenotype while FRβ^+^ TAMs exhibited an elongated cell shape with a M2-polarized pro-tumor profiles. IGF1, insulin-like growth factor 1; PM, peritoneal metastasis; TAMs, tumor associated macrophages.

TAMs are a key component of the TME which promote angiogenesis, tumor metastasis, and immune evasion (Mantovani et al., 2017). The serous cavity is a common metastatic site for a variety of malignant cancers, including colorectal cancer (Ceelen et al., 2020), gastric cancer (Song et al., 2019), ovarian cancer (Etzerodt et al., 2020; Xia et al., 2020), and lung cancer (Chow et al., 2021). When tumors metastasize into the peritoneal cavity, resident macrophages not only support tumor cell colonization and proliferation but also suppress the anti-tumor immune response (Figure 2).

### 3.1 The metastasis of tumors into peritoneal cavity relies on macrophages in omentum

The omentum is an adipose tissue layer containing certain milky spots (clusters of leukocytes) which are mainly composed of macrophages and B1 cells, resembling the cellular composition found in the peritoneal cavity. However, the phenotype of omental macrophages is different from peritoneal residential macrophages (Louwe et al., 2022). CD169 and LYVE1 are used to identify three omental macrophage subsets, in which the CD169^High^LYVE1^+^ subset can be further divided into four subpopulations based on the expression of CD163 and TIM4. Interestingly, many of the CD163^+^TIM4^+^ cells are embryonic origin and support the growth of metastatic ovarian cancer (Etzerodt et al., 2020). In peritoneal metastasis, the colonization of omentum is always associated with poor prognosis which provides a critical basement for tumor metastasis into the peritoneal cavity (Coccolini et al., 2013). Consistent with this observation, omentectomy inhibits tumor growth in the peritoneal cavity through a surgical procedure to remove the omentum (Etzerodt et al., 2020). Mechanically, ovarian cancer frequently colonizes the omentum which is observed passively through the milky spots (Rangel-Moreno et al., 2009), or fascinated by neutrophil extracellular traps (Lee et al., 2019), or recruited by the chemokines like IL-8 and CXCL12 (Nieman et al., 2011; Kasagi et al., 2016). Macrophages play an important role to assist the tumor cell colonization into omentum. It is reported that omental macrophages promote the migration and colonization of ovarian cancer cells to the omentum through the secretion of chemokine ligands that interact with chemokine receptor 1 (CCR1), and inhibition of CCR1 reduces ovarian cancer colonization (Krishnan et al., 2020). In addition, macrophages are supposed to form the immune suppressive microenvironment in the milky spots which are selectively invaded by tumor cells (Oosterling et al., 2006; Clark et al., 2013).

### 3.2 The promotion of tumor metastasis by peritoneal resident macrophages

#### 3.2.1 Support tumor growth and ascites formation

TAMs directly promote the development of metastases in ovarian cancer through the production of cytokines IL-6 (Isobe et al., 2015), vascular endothelial growth factor (VEGF) (Song et al., 2019), and transforming growth factor *β* (TGFβ) (Rodriguez et al., 2001), among others. In addition, insulin-like growth factor 1 (IGF1) expressed by TAMs increased the proliferation and migration of ID8 mouse ovarian cancer cells; while blockade of the IGF1 pathway in ID8 cells with an IGF1 neutralizing antibody effectively inhibited the ID8 caused tumor growth (Liu et al., 2018). Moreover, TAMs derived metabolites also contribute to the tumor growth. In peritoneal metastasis, GATA6^+^ residential macrophages elicit a fatty acid oxidation mediated an increase in oxidative phosphorylation (OXPHOS). Itaconic acid, a peritoneal resident macrophage specific metabolite which is produced by immune responsive gene 1 (IRG1) mediated catabolism of mitochondrial cis-aconitate, can promote tumor progression into the peritoneum. Knockdown of IRG1 significantly reduced peritoneal tumors with reductions in OXPHOS and ROS in TAMs and ROS-mediated MAPK activation in tumor cells (Weiss et al., 2018).

Patients with advanced peritoneal tumors often develop malignant ascites with fluid accumulation in the peritoneal cavity. The formation of peritoneal ascites is associated with increased vascular permeability and obstructed lymphatic drainage. The overexpression of VEGF causes the increased vascular permeability (Herr et al., 2012) accompanied with IL-6 and IL-10, as well as CXCL12 in ascites (Zeng et al., 2019). As the major source of VEGF, macrophages resided in peritoneal cavity promote the ascites formation in ovarian cancer and gastric cancer peritoneal metastasis (Song et al., 2019). The CSF1 antibody blockade and clodronate liposomes depletion inhibit peritoneal resident macrophage accumulation and prevent ascites formation in ovarian cancer (Robinson-Smith et al., 2007; Moughon et al., 2015). Ascites-derived spheroids in ovarian cancer facilitate tumor growth and progression. At early stages of transcoelomic metastasis of mouse epithelial ovarian cancer, M2 macrophage-like TAMs formed spheroids and secreted EGF, which upregulated αMβ2 integrin on TAMs and ICAM-1 on tumor cells to enhance association between tumor cells and TAMs, then to support tumor cell proliferation and migration (Yin et al., 2016).

#### 3.2.2 Facilitate immune evasion

In TME, TAMs have been summarized to be the dominant suppressive cells associated with immune evasion (Mantovani et al., 2017; Pathria et al., 2019). It is known that macrophages can directly target and eliminate the tumor cells. However, tumor expressed CD24 in ovarian cancer prevents macrophage-mediated phagocytic tumor clearance and promotes immune evasion by interacting with the inhibitory receptor sialic-acid-binding Ig-like lectin 10 (Siglec-10) expressed on TAMs (Barkal et al., 2019). Meanwhile, the presence of tumor infiltrating CD8^+^ T lymphocytes is highly associated with longer survival in ovarian cancer (Zhang et al., 2003; Peng et al., 2015). However, it is observed that the level of cavity resident macrophages is associated with reduced numbers of CD8^+^ T cells in pleural effusions and peritoneal ascites from patients with cancers. Mechanistically, TIM4 on the surface of cavity-resident macrophages interacts with its receptor phosphatidylserine (PS) upregulated on the surface of activated cytotoxic CD8^+^ T cells, which leads to CD8^+^ T cells sequestrated away from tumor targets and proliferation suppression by TIM4^+^ macrophages (Chow et al., 2021). In addition, canonical autophagy in peritoneal resident macrophages shows a suppressive effect on their IFNγ pathway activation (Wang et al., 2020). In mouse ID8 ovarian cancer metastasis, the depletion of autophagy related gene FIP200 in peritoneal TAMs induced T cell mediated anti-tumor response which may be due to the spontaneous IFNγ mediated immune activation in autophagy deficient TAMs (Xia et al., 2020). Hence, increased autophagy in peritoneal TAMs promotes ovarian cancer immune evasion.

### 3.3 The polarization of peritoneal tumor-associated macrophages remodeled by tumor cells

The plasticity of TAMs makes them polarized into two functional types, M1 (pro-inflammatory with anti-tumor activity) and M2 (anti-inflammatory with pro-tumor activity) macrophages, determined by their local TME (Murray et al., 2014; Muller et al., 2017). It is reported ovarian cancer cells polarize macrophages toward an M2 phenotype *in vivo* which gradually gain expression of M2-like marker genes, such as CD206, Arg1, and CD163, at 4–8 weeks after tumor injection (Yin et al., 2016). The homeobox A9 (HOXA9) expression in ovarian cancer cells stimulated chemotaxis of peritoneal macrophages and induced macrophages to acquire M2-like features (Ko et al., 2014). In a mouse ovarian metastatic model, tumor cells promote membrane-cholesterol efflux and depletion of lipid rafts from macrophages which promotes IL-4-mediated reprogramming but inhibits IFNγ-induced gene expression to accelerate tumor progression (Goossens et al., 2019). In addition, tumor-derived ubiquitin protein ligase E3 component N-recognin 5 (UBR5), an E3 ligase overexpressed in human ovarian cancer associated with poor prognosis, promotes TAMs recruitment and polarization *via* key chemokines and cytokines (Song et al., 2020). Meanwhile, M2-like TAM polarization could be repressed by sorbin and SH3 domain containing 2 (SORBS2) through stabilizing WAP four-disulfide core domain 1 (WFDC1) and IL-17D in ovarian cancer (Zhao et al., 2018). Furthermore, IFNγ secreted by tumor-infiltrating lymphocyte (TIL) is critical for M1 TAMs polarization. However, epigenetic silencing of CCL5 expression through DNA methylation in ovarian cancer cells leads to the TIL desertification and reduced IFNγ polarized M1 TAMs (Dangaj et al., 2019). Hence, the conversion of M2 TAMs to M1 TAMs provides an approach to improve the anti-tumor response by targeting TAMs.

### 3.4 The accumulation and discrepancy of peritoneal resident macrophage subsets in peritoneal metastasis

The accumulation of TAMs in peritoneal cavity reflects the development of peritoneal metastasis. Tumor derived factors, such as M-CSF and IL-4, promote the peritoneal residential macrophages self-proliferation or differentiation from monocytes. Due to the different ontogeny of peritoneal resident macrophages, the accumulation of peritoneal TAMs may originate from different precursors. In our work, the expression of TIM4 can be used to distinguish TIM4^+^ embryonic TAMs from TIM4^-^ monocytic TAMs which share very similar gene signatures with LPMs and SPMs in tumor free mice (Xia et al., 2020). Both TAM subsets expand in ID8 metastatic models. Further, we observe TIM4^+^ embryonic TAMs only depend on the self-proliferation while most of TIM4^-^ monocytic TAMs differentiate from infiltrated monocytes which were recruited by the tumor caused inflammation in peritoneal cavity. Compared to TIM4^-^ monocytic TAMs, TIM4^+^ embryonic TAMs express more OXPHOS genes and adapt to autophagy to support their survival in ovarian cancer metastasis. As the dominant TAMs in early tumor stage, TIM4^+^ embryonic macrophages promote the tumor metastasis (Xia et al., 2020). Similarly, surface expression of folate receptor (FR) *β* also can be used to identify two different peritoneal TAM subsets. FRβ^−^ TAMs display a round shape that is more monocytic in appearance and consistent with M1 phenotype. However, FRβ^+^ TAMs exhibits an elongated cell shape with a M2-polarized protumor profiles (Rodriguez-Garcia et al., 2021). In addition, the monocytic TAMs increase their percentage in total TAMs following the tumor growth and show some effects on tumor metastasis. At the advanced tumor stage, monocytic TAMs accumulate a lot from blood and elicit pro-tumor effect. For example, F4/80^Low^ SPMs can directly enhance ovarian cancer cell growth with expression of protumor and proangiogenic molecular mediators upregulated by IL-17 (Rei et al., 2014). Transcription factor ZEB1 expressed in F4/80^Low^ TAMs enhances tumor progression with the induction of a CCR2-MMP9-CCL2 positive loop between TAMs and cancer cells (Cortes et al., 2017).

Noticeably, the macrophages also reside within mesothelial membranes lining the peritoneal cavity which can be divided into LYVE1^High^MHC-II^Low^ and LYVE1^Low/−^MHC-II^High^ subsets. LYVE1^High^ macrophages predominantly originate from embryonic-derived progenitors and promote epithelial ovarian tumor growth. The LYVE1^High^ mesothelial macrophages express similar profiles with ovarian tumor-associated macrophages previously described in the omentum. These data reveal that the peritoneal compartment contains other two resident macrophage populations and that LYVE1^High^ mesothelial macrophages drive tumor growth (Zhang et al., 2021).

## 4 Targeting peritoneal resident macrophages to enhance anti-tumor immunotherapy

Harnessing the immune system provides an important approach to combating cancer. Several strategies have been developed to manipulate the immune response, including immune checkpoint blockade (ICB), chimeric antigen receptor T cells (CAR T cells), dendritic cell vaccines, and cytokine therapies. Peritoneal resident macrophages assist tumors to suppress the immune response which should be a good target to improve the effect of immunotherapy (Duan and Luo, 2021). Several strategies have been developed to target the TAMs in peritoneal cavity:

### 4.1 Myeloid checkpoint blockade

Blocking the PD-1/PD-L1 or CTLA-4 pathways with antibodies relieves the cytotoxic function of T cells to control the tumor growth (Zou et al., 2016). However, patients with peritoneal metastasis are hardly responsive to the PD-1/PD-L1 blockade indicating that there are other checkpoints involved to hinder the effect of ICB in peritoneal metastasis. Along with the immune checkpoints on T cells, several checkpoints that are mainly associated with macrophages have been discovered. TIM4^+^ cavity TAMs sequester and impair proliferation of CD8^+^ T cells under the ICB treatment through the interaction between TIM4 and PS. Hence, TIM4 blockade abrogates this sequestration and proliferation suppression and enhances anti-tumor efficacy in models of anti-PD-1 therapy and adoptive T cell therapy in mice (Chow et al., 2021). CD47 on the tumor surface acts as a “don’t eat me” signal and prevents macrophage-mediated phagocytosis through its direct interaction with signal regulatory protein-*α* (SIRPα). Blockade of CD47 signaling by using targeted monoclonal antibodies restores the phagocytic capacity of TAMs which enables macrophage phagocytosis of ovarian cancer cells (Willingham et al., 2012; Liu R. et al., 2017). Furthermore, a phase I trial of an anti-CD47 antibody Hu5F9-G4 demonstrated two patients with ovarian/fallopian tube cancers had partial remissions for 5.2 and 9.2 months (Sikic et al., 2019). In addition, the CD24/Siglec-10 signal also provide a target for restoring the phagocytosis of peritoneal resident TAMs in ovarian cancer (Barkal et al., 2019).

### 4.2 Tumor-associated macrophages depletion

Emerging evidence show the loss of TAMs destroy the suppressive TME and promote anti-tumor immune response. Targeting the differentiation and proliferation of TAMs always influence their population. Administration of GW2580, an inhibitor of CSF1 receptor, reduced infiltration of M2-like macrophages and dramatically decreased ascites volume in the late stages of ovarian cancer metastasis (Moughon et al., 2015). Depletion of GATA6 induces peritoneal residential macrophage apoptosis which dramatically decreases their numbers (Gautier et al., 2014; Rosas et al., 2014). Blocking the induction of GATA6 or inhibiting its transcriptional activity should strongly impair the identity of peritoneal resident macrophages and reduce their numbers. For example, retinoid X receptors (RXRs) control mouse serous-macrophage identity by regulating chromatin accessibility and the transcriptional regulation of canonical macrophage genes, partially through GATA6. RXRs deficiency impairs neonatal expansion of the LPM pool and reduces the survival of adult LPMs through excess lipid accumulation. Depletion of RXR diminishes LPMs accumulation in ovarian cancer and strongly reduces tumor progression in mice which indicates targeting RXR signaling may improve ovarian cancer outcomes *via* interfering the maintenance of the serous macrophage pool (Casanova-Acebes et al., 2020). In addition, the peritoneal resident TAMs highly relied on the autophagy for survival compared to the migrated TAMs. Autophagy can be used as a target to selectively reduce the TIM4^+^ residential TAMs and tumor growth (Xia et al., 2020).

Some artificial materials are developed to destroy the peritoneal TAMs. Clodronate liposome elicits toxic effects on macrophages *via* phagocytosis. The administration of clodronate liposome reduces the number of peritoneal TAMs and metastasis in ovarian cancer (Etzerodt et al., 2020; Xia et al., 2020). CAR T cells are a type of immunotherapy that involves T cells genetically modified to express receptors that recognize cancer-specific antigens (Kershaw et al., 2013). Interestingly, Rodriguez-Garcia et al. (2021) developed mouse and human FRβ-specific CAR T cells which recognize and deplete the FRβ^+^ TAMs in ovarian cancer metastasis. Furthermore, pre-treatment of the TME with anti-TAM CAR T cells improves the efficacy of tumor-specific CAR T cells against ovarian cancer metastasis. Similarly, G5-methotrexate (G5-MTX) nanoparticles restrict tumor growth by targeting and depleting the FRβ^+^ TAMs in ascites models of ovarian cancer (Penn et al., 2018).

### 4.3 Metabolic remodeling

It is well known that aggressively proliferated tumor cells consume amount of nutrients and fulfill the TME with their metabolic products which crosstalk with TAMs and form immune suppressive TME (Chang et al., 2015). This immunosuppressive remodeling seems not because of direct nutrient competition since cell-intrinsic programs drive the preferential acquisition of glucose and glutamine by immune and cancer cells (Reinfeld et al., 2021). Cancer cells show the highest uptake of glutamine which is not only their fuel but also drives M2-like macrophage polarization *via* epigenetic modifications (Liu P. S. et al., 2017). In addition, certain tumor derived metabolites function to promote the polarization of M2 TAMs phenotype, including lactic acid (Colegio et al., 2014), succinate (Wu et al., 2020), and so on. Hence, alteration of the tumor or macrophage metabolism provides an approach to convert the anti-inflammatory TAMs into pro-inflammatory TAMs. For example, genetic depletion of glutamine synthetase skews macrophages toward an M1-like phenotype and inhibits tumor metastasis (Palmieri et al., 2017).

In the context of peritoneal grafted cancer models, it is observed that the fatty acid oxidation (FAO), a way to break down a fatty acid into acetyl-CoA, increase in peritoneal resident macrophages (Weiss et al., 2018). Lipid metabolism is critical for the identity and homeostasis of LPMs and RXR-deficient peritoneal TIM4^+^ LPMs leads to lipid accumulation and apoptosis (Casanova-Acebes et al., 2020). Considering the LPMs represent the dominant peritoneal TAMs, interrupting their lipid metabolism may have the effect to control the ovarian cancer metastasis by inducing their apoptosis. Moreover, hyaluronic acid secreted by ovarian cancer cells promotes plasma membrane cholesterol efflux in TAMs *via* binding to CD44, which enhances IL-4 receptor signaling *in vitro* and *in vivo* associated with reduced intracellular cholesterol, finally promoting the expression of the typical M2 marker Arg1 while inhibiting proinflammatory IL-12 expression (Goossens et al., 2019). Deletion of cholesterol efflux genes ATP-binding cassette transporter A1 (ABCA1) and ATP-binding cassette transporter G1 (ABCG1) in peritoneal TAMs has been found to significantly impair tumor progression (Goossens et al., 2019). In addition, mTORC2 is important for the metabolic reprogramming of tissue-resident macrophages (Oh et al., 2017) and TAMs (Huang et al., 2016), so mTORC2 can be a potential target to remodel TAMs from an anti-inflammatory to an anti-tumor phenotype. Hence, metabolic rewiring of peritoneal TAMs should be an alternative strategy to enhance the anti-tumor immunity.

## 5 The human counterpart of peritoneal resident macrophages in cavity metastasis

Human studies on peritoneal macrophage populations divide them into three distinct subsets based on the expression of CD14/CD16 (CD14^++^CD16^−^, CD14^++^CD16^+^, and CD14^High^CD16^High^). The CD14^High^CD16^High^ subset represents the mature phenotype of steady-state human resident peritoneal macrophages based on the expression of GATA6, and other resident macrophage markers, such as CD206 and Slan (Ruiz-Alcaraz et al., 2016; Ruiz-Alcaraz et al., 2018). Consistent with mouse models, emerging evidence demonstrate that human peritoneal resident macrophages promote human cancer metastasis into cavity. For example, gastric cancer (GC) patients with peritoneal metastasis had increased levels of alternatively activated macrophages in the peritoneum compared to those without dissemination. Macrophages in the peritoneal cavity produce EGF and VEGF to enhance the angiogenesis in GC patients bearing peritoneal metastasis. Hence, patients bearing more macrophages in the peritoneum had a poorer prognosis (Song et al., 2019). The ascites provides a convenient way to isolate high amount of pure human macrophages which can be analyzed through their transcriptome and proteome. It is reported TAMs from human ovarian carcinoma ascites shared similar character with peritoneal resident macrophages, but not monocyte-derived macrophages. The elevated signature genes in human TAMs are highly related to extracellular matrix (ECM) remodeling which indicates the role for TAMs in cancer cell invasion and ovarian cancer progression (Finkernagel et al., 2016). Meanwhile, two subgroups of ascites macrophages have been identified in ovarian cancer patients. Subgroup A has a high expression of pro-tumor markers (CD163, PCOLCE2, IL-6) related to immune suppression and ECM remodeling while subgroup B has a low expression of pro-tumorigenic and immunosuppressive markers with an upregulation of genes linked to interferon signaling (Adhikary et al., 2017). Furthermore, the expression of complement receptor of the immunoglobulin superfamily (CRIg) and CCR2 have been used to define two phenotypically and functionally distinct human peritoneal macrophage subpopulations (Irvine et al., 2016). CRIg^High^ cells are transcriptionally, metabolically, and functionally similar with the mouse F4/80^High^ resident peritoneal macrophages in cirrhosis (Irvine et al., 2016) and ovarian cancer patients (Xia et al., 2020).

## 6 Conclusion

As the dominant myeloid cells infiltrating TME, TAMs promote immune evasion through multiple routes, including triggering of inhibitory immune checkpoints in T cells. Understanding the ontogeny and modulation of those immunosuppressive cells is critical for overcoming their disadvantages. Different from monocytic macrophages, peritoneal resident macrophages originate from embryonic precursors which locate in peritoneal cavity during development and can self-maintain locally throughout life with tissue-specific levels of replacement by circulating precursors (Bain et al., 2016). After tumor cells infiltrate into the peritoneal cavity, peritoneal resident macrophages become the primary macrophages surrounding the tumor cells and promote the tumor growth at the early stage. Meanwhile, inflammatory macrophages differentiated from blood monocytes gradually increase in numbers as the tumor progresses, contributing to peritoneal metastasis at the late stage. Determining whether peritoneal resident TAMs are predictive biomarkers for early peritoneal metastasis is important for personalized patient care in clinical applications.

Targeting the peritoneal resident TAMs elicits anti-tumor immune response and controls tumor metastasis. The tumor spheroids, angiogenesis, and immune suppression microenvironments supported by peritoneal TAMs promote tumor survival and growth. Understanding the differentiation, polarization, and metabolism of peritoneal TAMs is beneficial for exploring different approaches to reduce or remodel TAMs. The development of new technologies, such as CAR-macrophage and CAR-T targeting to macrophages, extend the potential immunotherapy by modulating peritoneal macrophages. Given the peritoneal resident macrophages can be recruited to injured organs in peritoneal cavity, it is still unknown if the peritoneal resident macrophages have effects on the orthotopic tumor growth and initiate the tumor transformation and metastasis. In the future, more challenges need to be addressed to manipulate TAMs in peritoneal metastasis, including 1) to understand the metabolic consumption of peritoneal TAMs and the pro-tumor effects of TAMs related metabolites; 2) to discover peritoneal specific transcriptional factors and surface markers to distinguish pro- and anti-tumoral peritoneal TAM subsets; 3) to prevent the clearance of the tumor neo-antigens by peritoneal TAMs; 4) to reduce the interference of peritoneal TAMs in antigen presentation; 5) to explore peritoneal macrophage related epigenetic regulations of their profiles; 6) to avoid peritoneal TAMs mediated immunotherapy resistance. In summary, peritoneal resident macrophages promote tumor metastasis into peritoneal cavity and can be targeted to enhance T-cell immunity, to modify polarization of TAMs, and to enhance phagocytosis of cancer cells with or without other therapies.
